# Supplementary material for: Atomic cobalt as an efficient electrocatalyst in sulfur cathodes for superior room-temperature sodium-sulfur batteries
Source: Nat Commun. 2018 Oct 4;9:4082. doi: 10.1038/s41467-018-06144-x (PMC6172263; doi:10.1038/s41467-018-06144-x)
Supplement: Supplementary file 1 — Supplementary Information [file 41467_2018_6144_MOESM1_ESM.pdf]

Supplementary Information for

Atomic cobalt as an efficient electrocatalyst in sulfur cathodes for superior room-temperature sodium-sulfur batteries

Zhang et al

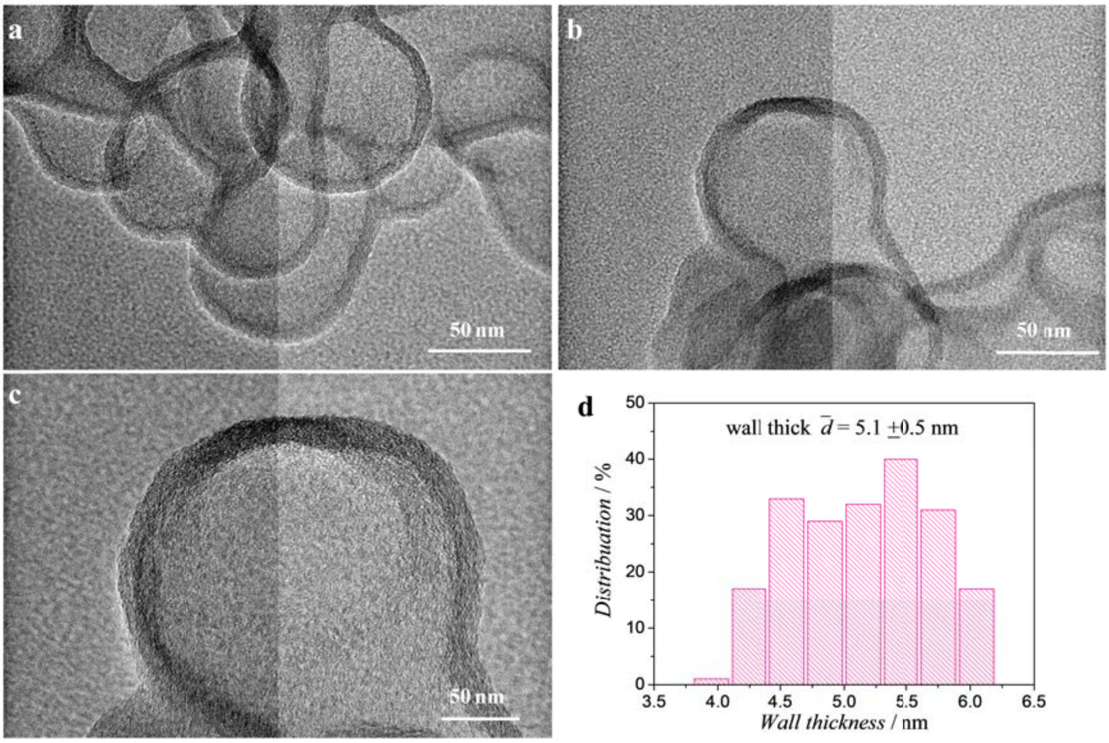

Supplementary Figure 1. a-c, TEM images of HC. **d,** Histogram showing wall thickness distribution based on a count of 200 wall thick in the sample areas.

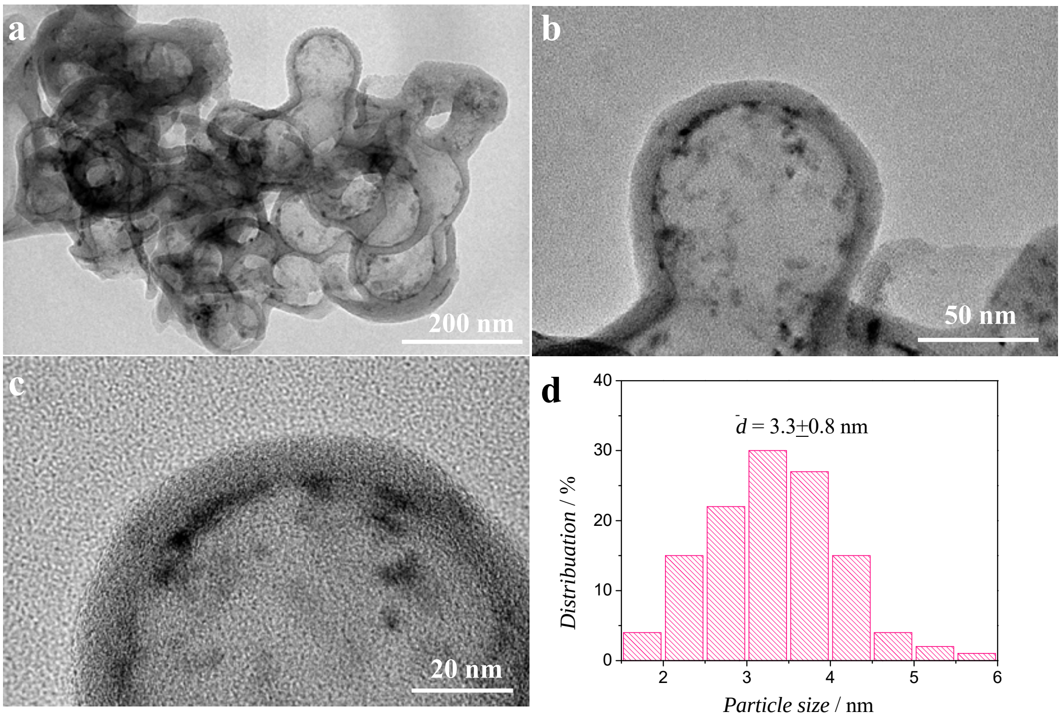

Supplementary Figure 2. a-c, Low and high magnification TEM images of Co-HC. **d,** Histogram showing Co nanoparticles distribution based on a count of 200 Co nanoparticles in the sample areas.

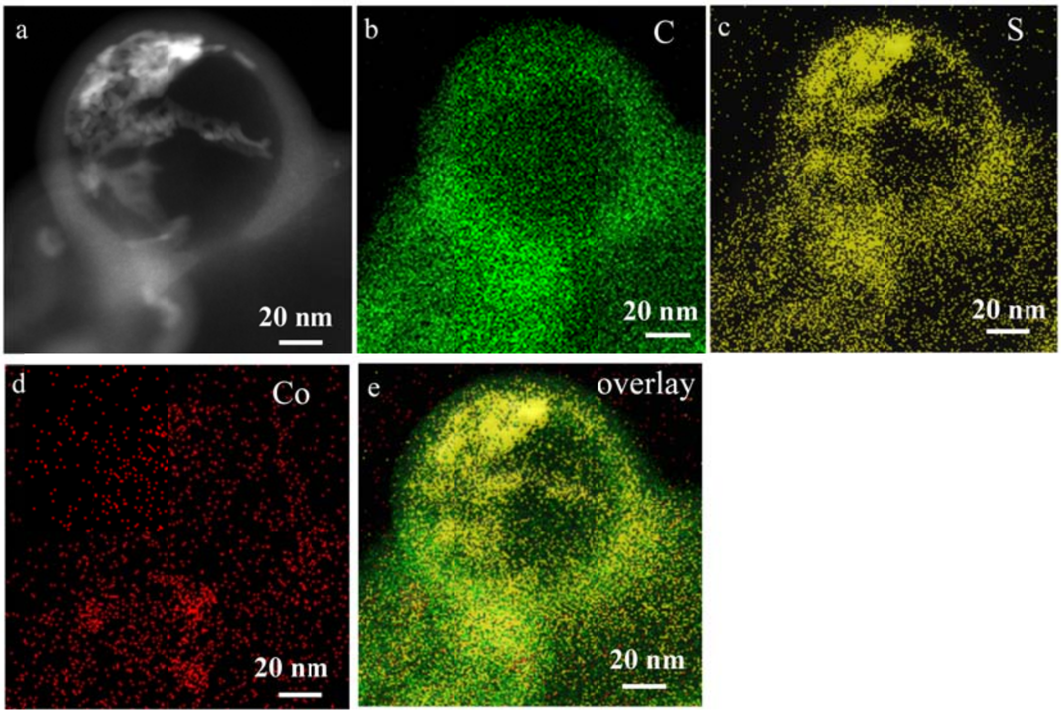

Supplementary Figure 3. a, HADDF-STEM images of S/Co-HC. **b-e,** elemental mapping of S/Co-HC.

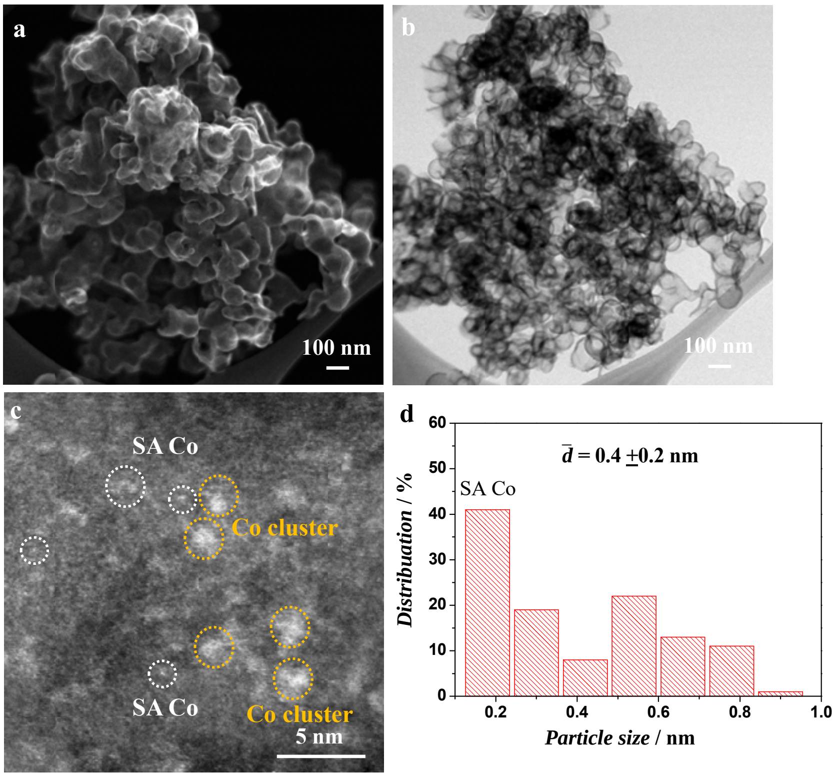

Supplementary Figure 4. a, SEM, **b,** TEM and **c,** HADDF-STEM image of S@Co_n-HC. **d,** Histogram showing S@Co_n-HC distribution based on a count of 200 clusters in the sample areas.

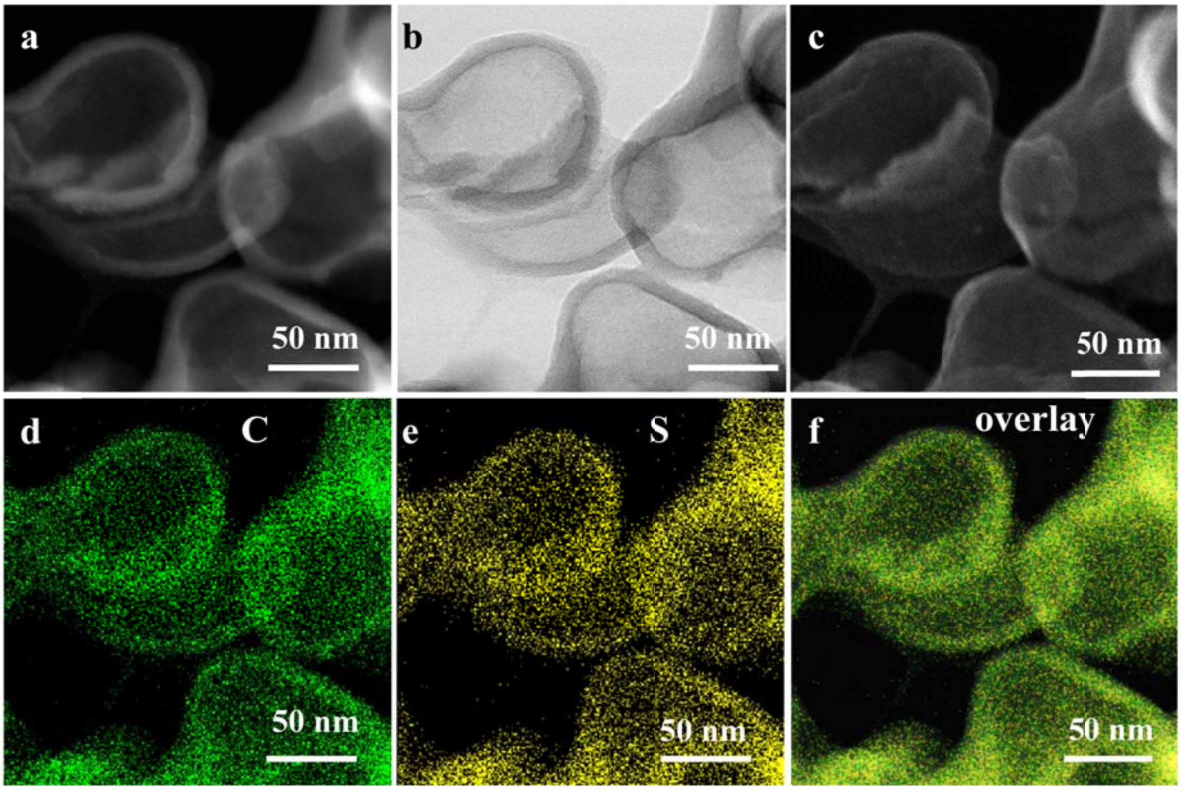

Supplementary Figure 5. a, STEM image, **b,** TEM image, **c,** SEM image and **d-f,** elemental mapping of S@HC.

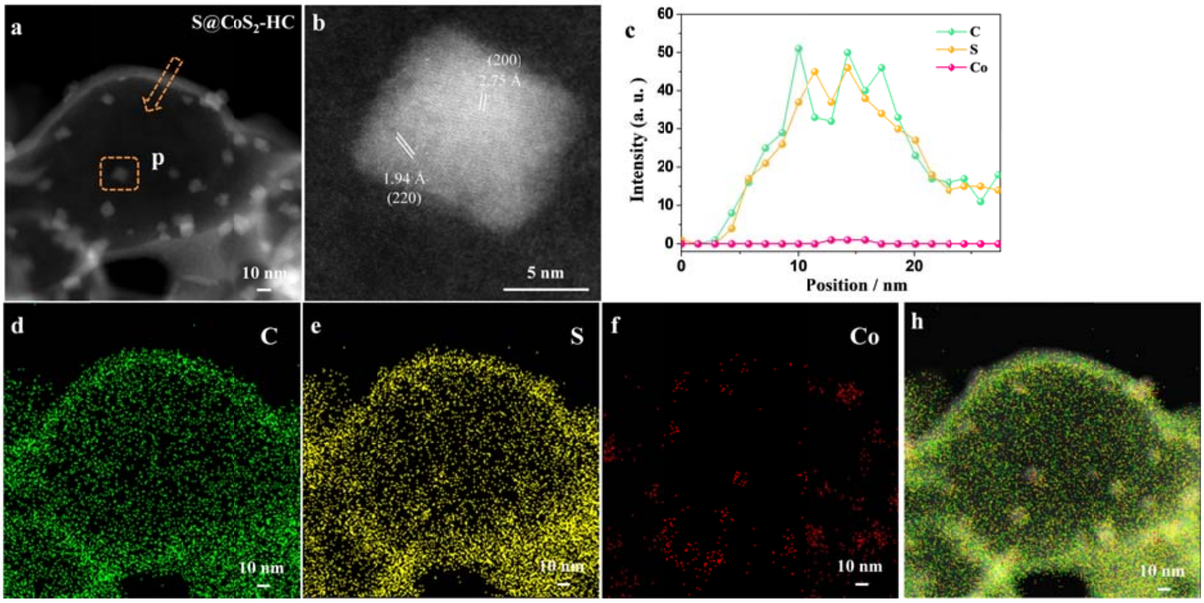

Supplementary Figure 6. a-b, HAADF-STEM images of S@CoS_2-HC. **c,** line-profile analysis of the indicated of line in **a. d-h** elemental mapping of S@CoS_2-HC.

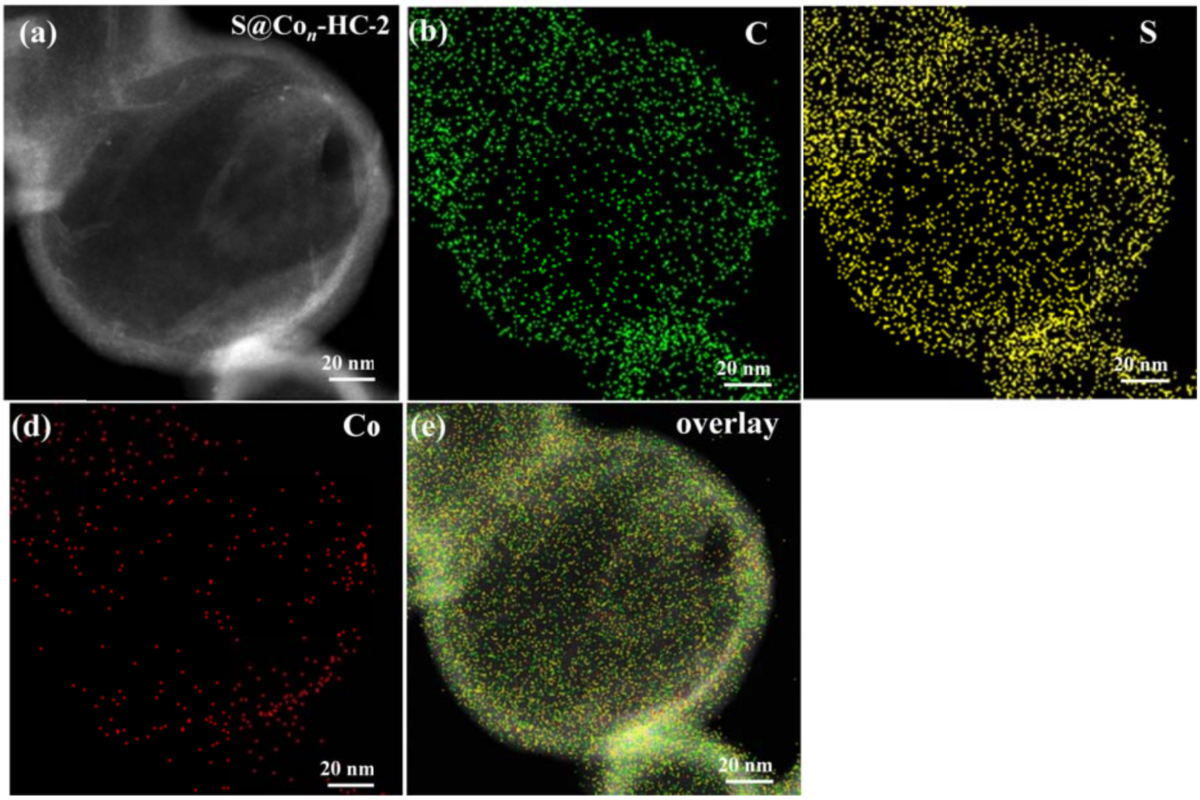

Supplementary Figure 7. a, HADDF-STEM images of S@Co$_x$-HC. **b-e,** elemental mapping of S@Co$_n$-HC.

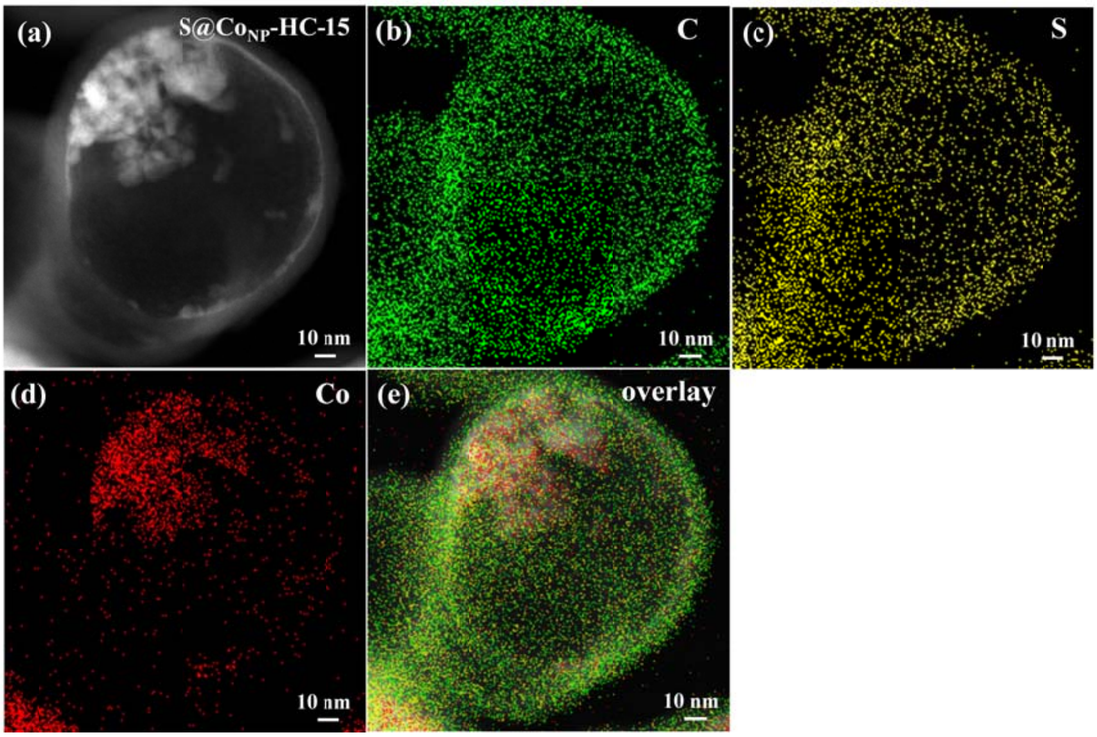

Supplementary Figure 8. a, HADDF-STEM images of S@Co$_{NP}$-HC. **b-e,** elemental mapping of S@Co$_{NP}$-HC.

Supplementary Note 1

For the S@Co_n-HC, the ICP-OES result demonstrates that the Co content is 7.06 wt. %, which is prepared form 10 wt. % of $CoCl_2$. For optimization, 5% and 20% of $CoCl_2$ are applied via the same synthesis procedures. ICP-OES results show that the weight content of Co in these two sample are 2.33% and 15.02%, named as S@Co_n-HC-2 and S@Co_{NP}-HC-15 respectively. The HAADF-STEM with elemental mapping of S@Co_n-HC-2 in Supplementary Fig. 7 demonstrate the similar morphologies and components to that of the S@Co_n-HC. Atomic Co is well confined in the carbon shells, and sulfur is also well dispersed on the carbon shell; meanwhile, the Co elemental mapping in Supplementary Fig. 8 also indicates that the content of Co is lower than S@Co_n-HC. However, the HAADF-STEM results of S@Co_{NP}-HC-15 in Supplementary Fig. 8 indicate that Co nanoparticles are formed at this high Co content. The TGA results of these different Co content cathode materials in Supplementary Fig. 9 indicate that the S contents in S@Co_n-HC-2, S@Co_n-HC, and S@Co_{NP}-HC-15 are ~ 36 %, 47 %, and 47 %, respectively. The low S loading ratio of S@Co_n-HC-2 (36%) indicates that high atomic Co content in HC is favourable to capture S and enhance S loading amount; it is noteworthy that the sulfur loading ratio of S@Co_{NP}-HC-15 equal with that of S@Co_n-HC, demonstrating that only simply increasing the Co content can not enhance the sulfur loading.

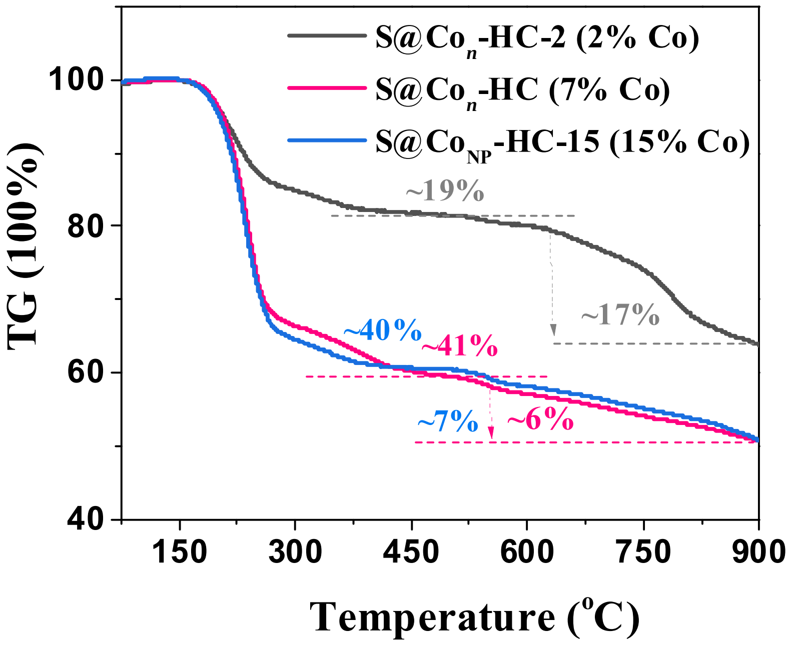

Supplementary Figure 9. TGA of S@Co_n-HC-2, S@Co_n-HC and S@Co_{NP}-HC.

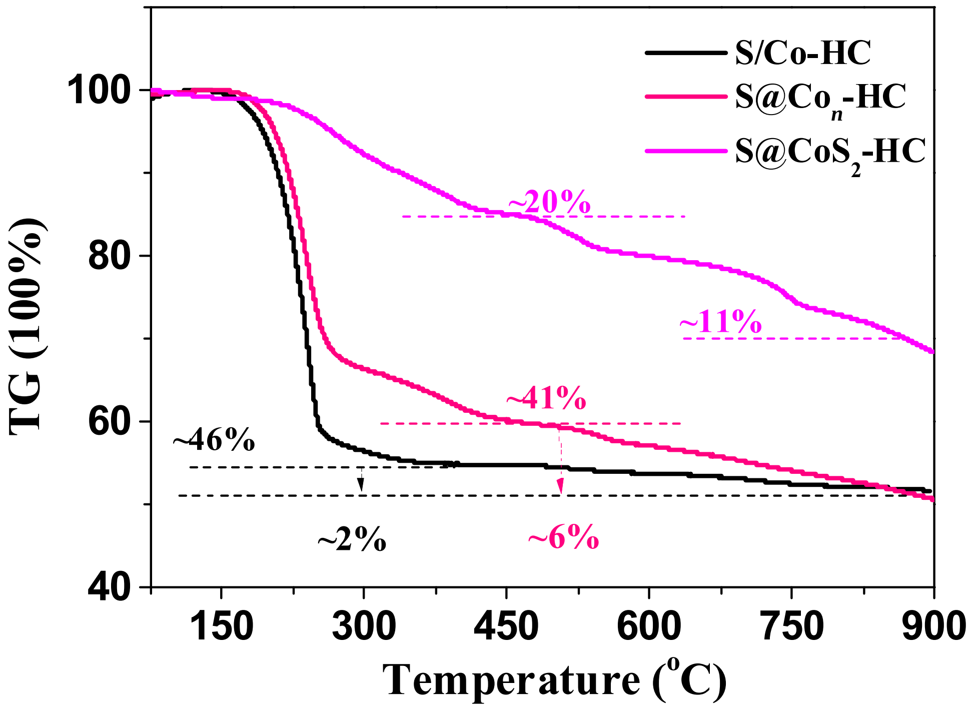

Supplementary Figure 10. Thermogravimetry (TGA) curves of S/Co-HC, S@Co$_n$-HC, and S@CoS$_2$-HC.

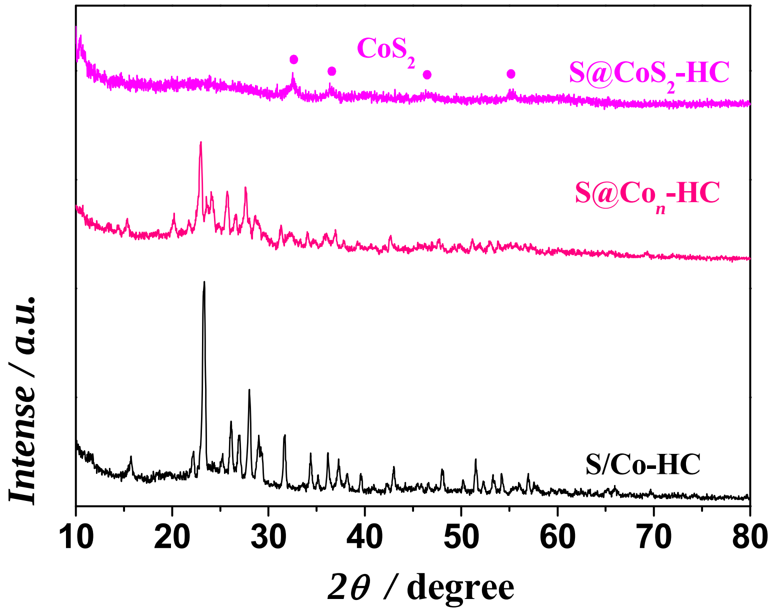

Supplementary Figure 11. XRD curves of S/Co-HC, S@Co$_n$-HC, and S@CoS$_2$-HC.

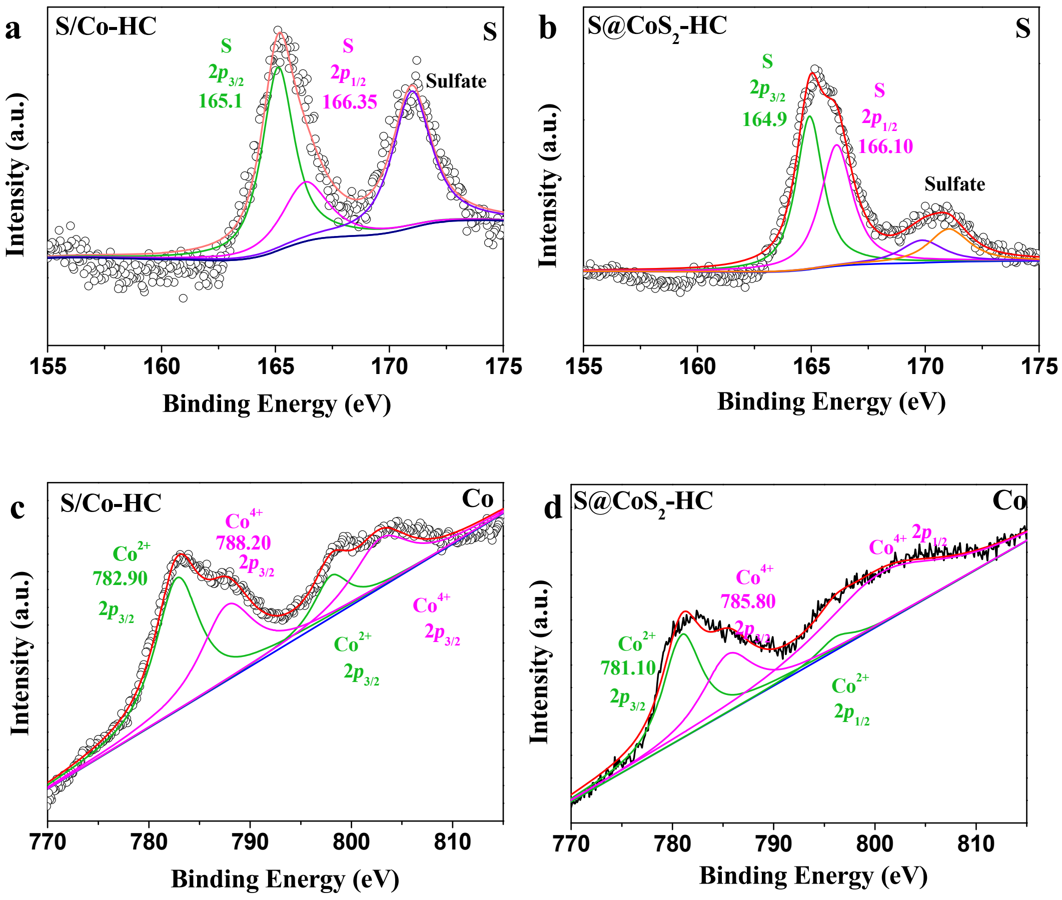

Supplementary Figure 12. a-c, XPS S 2p spectra and **d-f,** Co 2p spectra of S/Co-HC and $S@CoS_2$-HC.

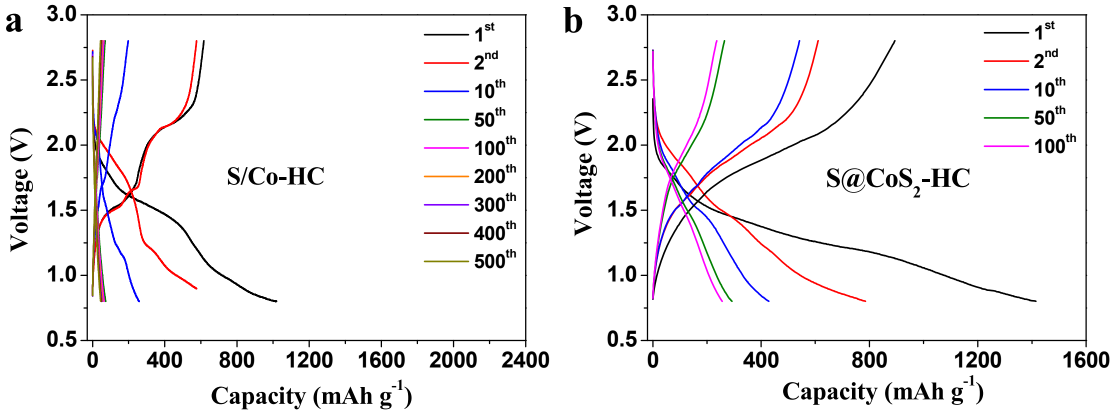

Supplementary Figure 13. Discharge/charge voltage profiles of RT-Na/S cell of a, S/Co -HC, b, $S@CoS_2$-HC at selected cycles at 100 mA g^{-1}.

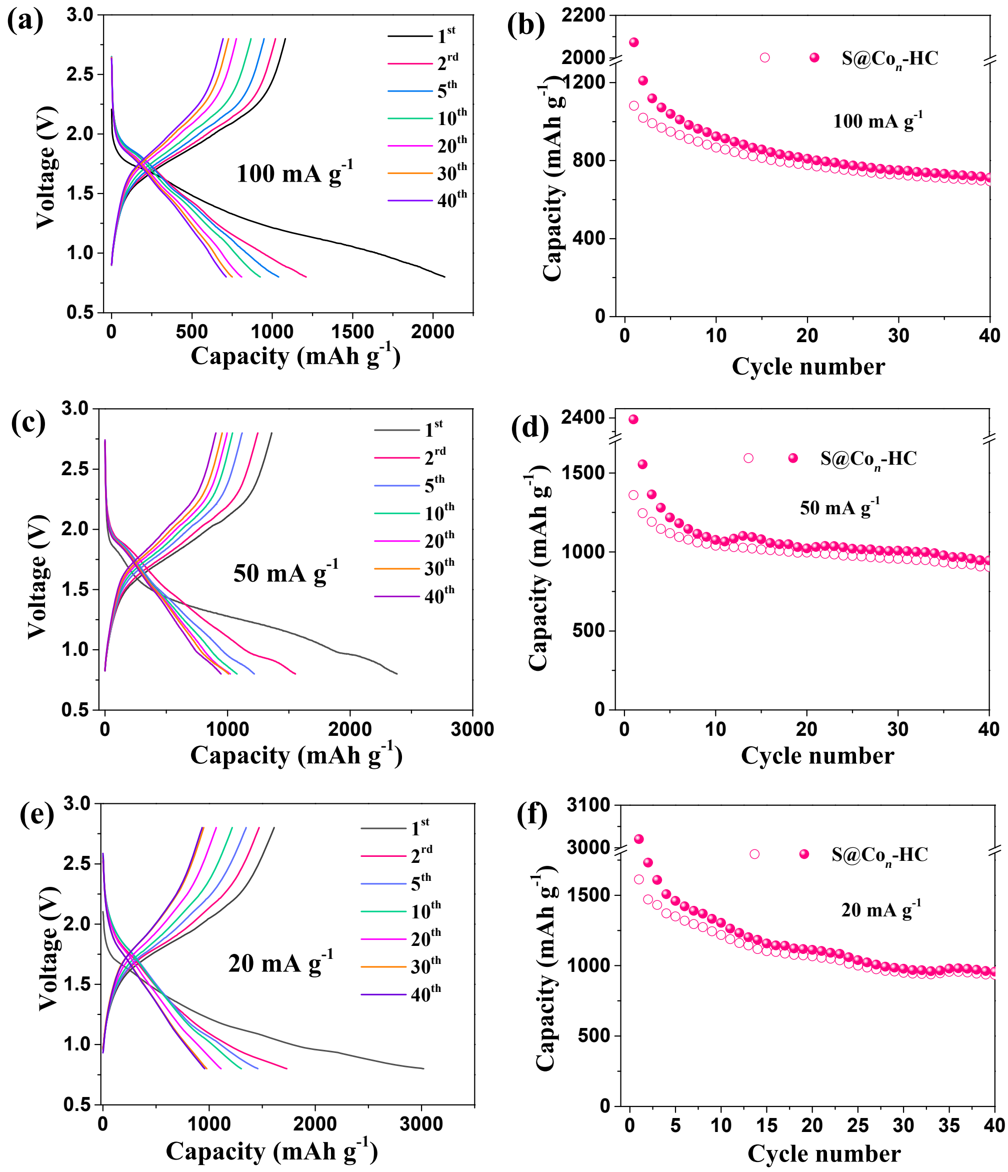

Supplementary Figure 14. Discharge/charge curves of S@Co$_n$-HC at 100 mA g^{-1} (a), 50 mA g^{-1} (c) 20 mA g^{-1} (e). Cycle performance S@Co$_n$-HC at 100 mA g^{-1} (b), 50 mA g^{-1} (d) 20 mA g^{-1} (f).

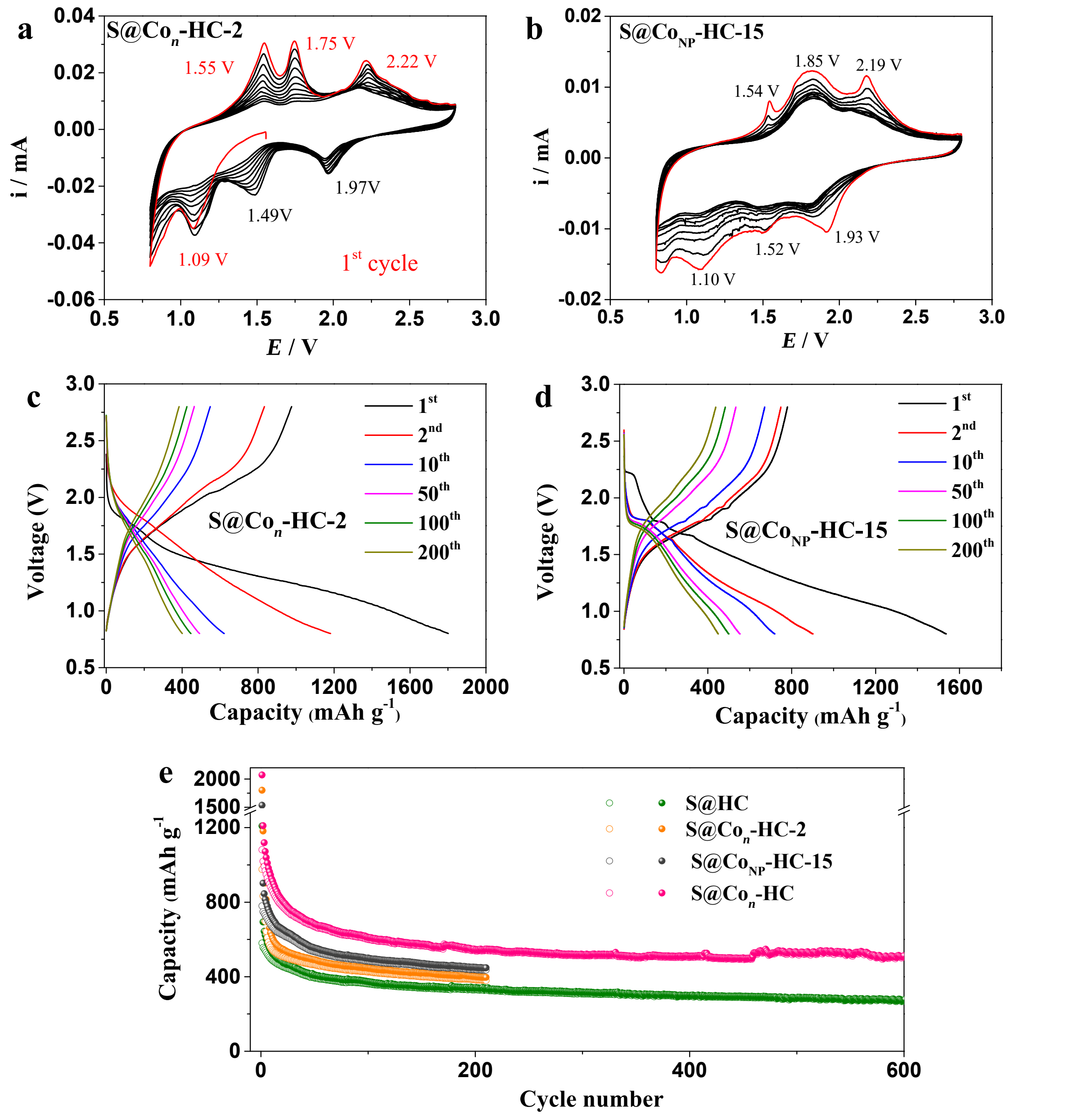

Supplementary Figure 15. a and b, Cyclic voltammograms of S@Co$_n$-HC-2 and S@Co$_{NP}$-HC-15 within the voltage window of 0.8 - 2.8 V at a scan rate of 0.1 mV s^{-1}. **c and d**, Discharge/charge curves of S@Co$_n$-HC-2 and S@Co$_{NP}$-HC-15 at 100 mA g^{-1}. **e,** Cycle performance of S@Co$_n$-HC-2, S@Co$_{NP}$-HC-15, S@Co$_n$-HC and S@HC at 100 mA g^{-1}.

Supplementary Note 2

To investigate the impact of Co contents on electrochemical performance, their CVs and discharge/charge profiles of the 1st, 2nd, 10th, 50th, 100th and 200th cycles at 100 mA g^{-1} of S@Co$_n$-HC-2, S@Co$_n$-HC and S@Co$_{NP}$-HC-15 cathode materials are shown in Supplementary Fig. 15. The CV of S@Co$_n$-HC-2 has three peaks at 1.97, 1.49, and 1.09 V

during cathodic cycling and three peaks at 1.55, 1.75, and 2.22 V in the anodic scan, indicating that S is reduced in an orderly manner to Na_2S_x, Na_2S_4, and Na_2S. It is interesting that CV peaks of S@Co_{NP}-HC-15 are almost the same, indicating that they may go through the same mechanism. The RT-Na/S@Co_n-HC-2 cell shows three long plateaus from 1.91 to 1.50 V, 1.50 to 1.10 V and 1.10 to 0.8 V during the initial discharge process, corresponding to its CV results. Meanwhile, the S@Co_{NP}-HC-15 presents four plateaus from 2.28 to 2.20 V, 1.81 to 1.77 V, 1.71 to 1.66 V, and 1.66 to 0.8 V during the initial discharge process. The lower potential plateaus of S@Co_n-HC-2 in the initial cycle may be attributed to that the atomic Co has a stronger ability to immobilize S than Co nanoparticles, even the Co context of S@Co_n-HC-2 (2.33%) is quite lower than that of S@Co_{NP}-HC-15 (15.02%). The initial reversible capacity of S@Co_n-HC-2 and S@Co_{NP}-HC-15 are 976 and 780 mAh g^{-1}, respectively; meanwhile they retain the reversible capacity of 383 and 438 mAh g^{-1} after 200 cycles. Significantly, the S@Co_n-HC processes the best performance among these cathode materials, as shown in Supplementary Fig. 15.

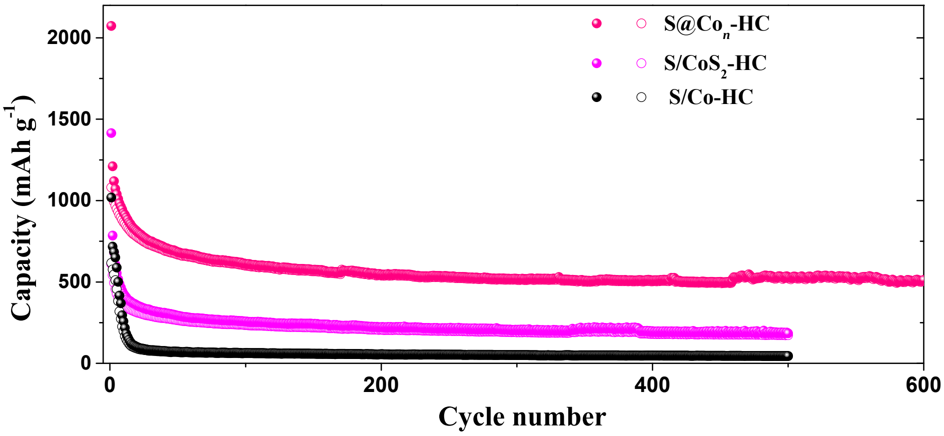

Supplementary Figure 16. Cycling performance at 100 mA g^{-1} of S/Co-HC, S@Co_n-HC and S@CoS_2-HC.

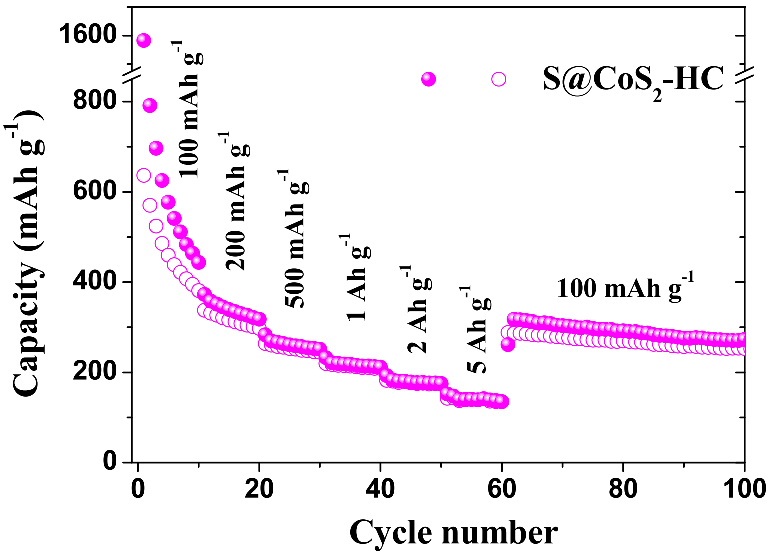

Supplementary Figure 17. Rate performance of S@CoS_2-HC.

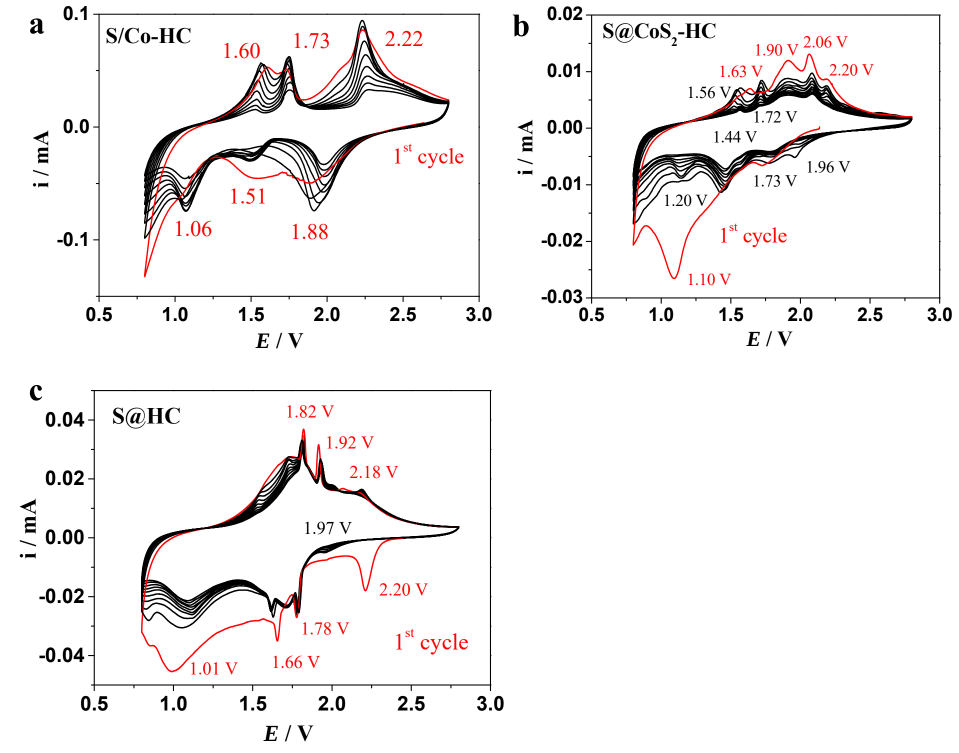

Supplementary Figure 18. Cyclic voltammograms of **a,** S/Co-HC, **b, S@**CoS_2-HC, and **c,** S@HC in RT-Na/S cell for 10 cycles within the voltage window of 0.8 - 2.8 V at a scan rate of 0.1 mV s^{-1}.

Supplementary Note 3

CVs of S/Co-HC, S@CoS_2-HC and S@HC are shown in Supplementary Fig. 18. It is interesting that the CV shape of S/Co-HC also has three peaks at 1.88, 1.51, and 1.06 V during cathodic cycling and three peaks at 1.60, 1.73, and 2.22 V in the anodic scan, indicating that S is reduced in an orderly manner to Na_2S_x, Na_2S_4, and Na_2S. The RT-Na/S@HC cell presents four peaks at around 2.20, 1.78, 1.66, and 1.01 V during the initial cathodic scan. Specifically, the peak at 2.20 V is in agreement with a solid-liquid transition from S to dissolved long-chain polysulfides (Na_2S_x, $4 < x < 8$); the peak at 1.78 V could be assigned to the formation of Na_2S_4; the 1.66 V peak matches short-chain polysulfides (Na_2S_y, $1 < y < 4$); and the peak at 1.01 V also could be attributed to formation of Na_2S[40,41]. The anodic scan of RT-Na/S@HC cell has three peaks (1.82, 1.92, and 2.20 V), corresponding to the formation of Na_2S, Na_2S_4, and long-chain polysulfides. Interestingly, the CV of S@CoS_2-HC also has four peaks, which means that it may also go through Na_2S_x, Na_2S_4, Na_2S_m, and Na_2S.

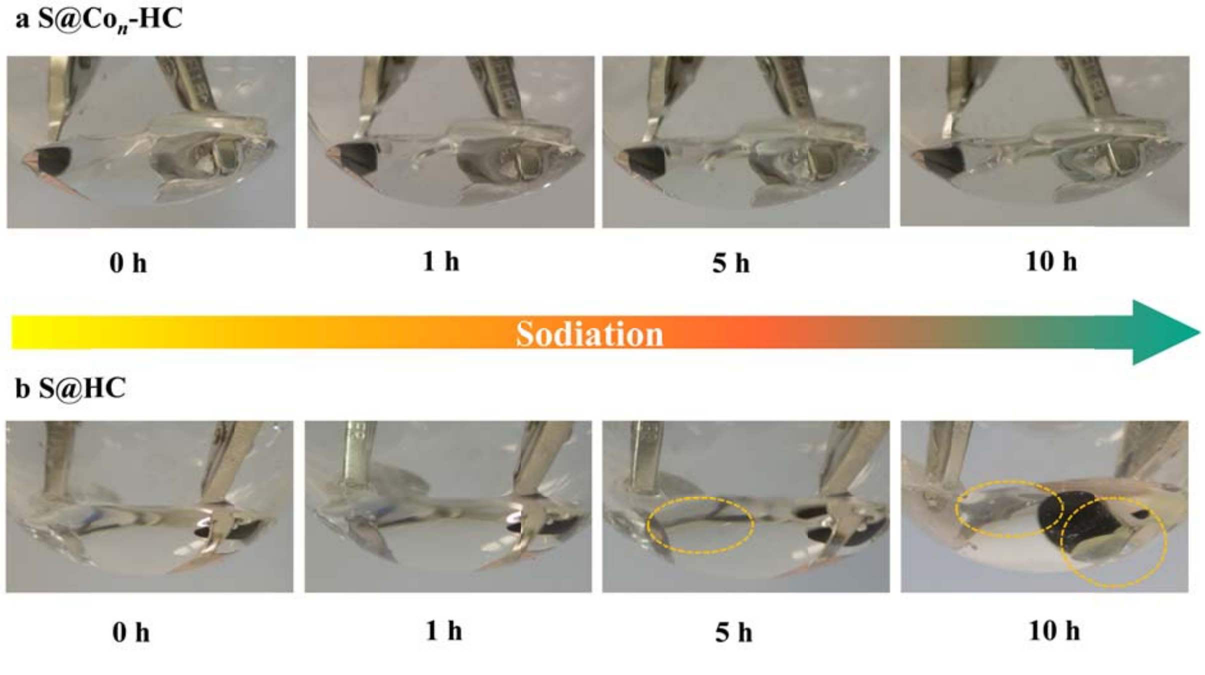

Supplementary Figure 19. Visual examination of polysulfides entrapment of (a) S@Co_n-HC and (b) S@HC at specific sodiation depths.

In order to make sure S host, the Co_n-HC, in S@Co_n-HC is inactive in the RT/Na-S batteries, we have studied the electrochemical performance of the plain matrix, in which S is removed from S@Co_n-HC to form Co_n-HC. The removing S experimental process is following:

30 mg S@Co_n-HC was dispersed in 30 mL CS_2 solution. The mixture was ultrasonicated for 2 h, followed by washing by ethanol, acetone and DI water with several times, respectively. Finally, the obtained black powder was heated overnight in an oven at 80 ^{o}C for 12 h.

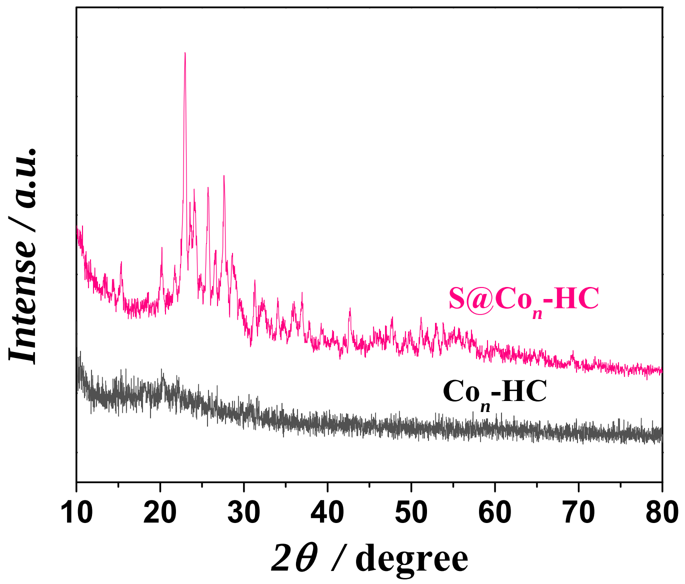

Supplementary Figure 20. XRD of Co_n-HC and S@Co_n-HC.

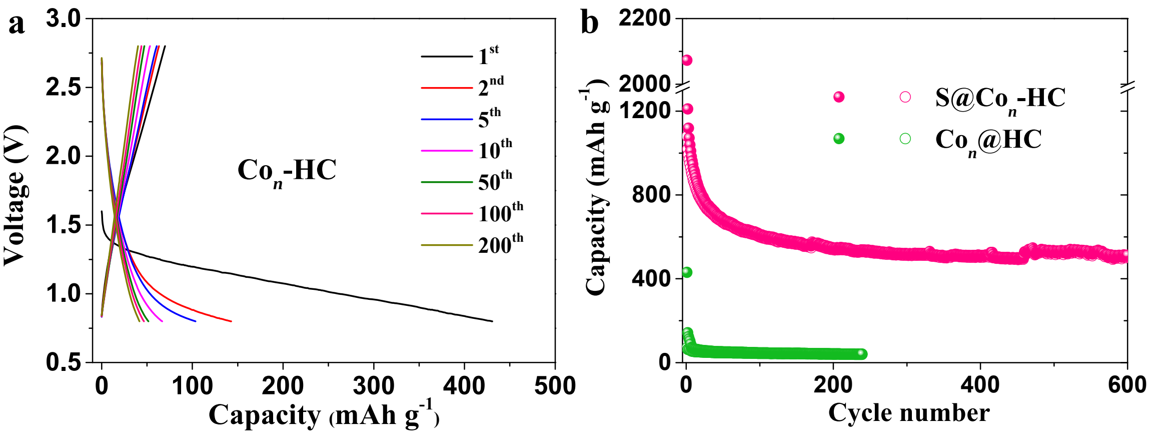

Supplementary Figure 21. a, discharge/charge curves and **b,** cycle performance of Co_n-HC and S@ Co_n-HC at 100 mA g^{-1}.

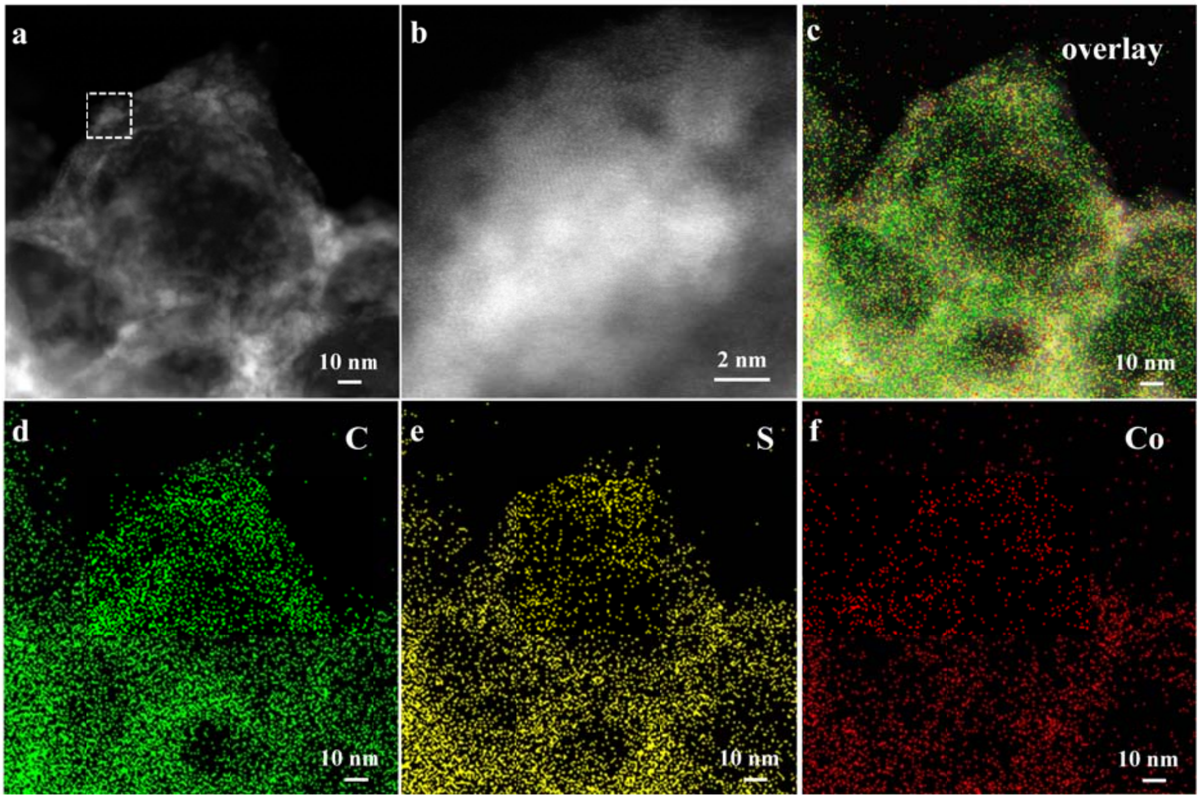

Supplementary Figure 22. **a-c**, STEM image of S@Co$_n$-HC after 600 cycles, with the corresponding elemental mapping for **(d)** C, **(e)** S, and **(f)** Co.

The compositional and morphological changes of S@Co$_n$-HC after 600 cycles shown in Supplementary Fig. 22 also demonstrate that the HC could keep its morphology, while Co agglomerates to larger nanoparticles. Nevertheless these larger nanoparticles are also supported on the carbon shell, which still has the ability to alleviate the "shuttle effect", suggesting that it could maintain higher capacity.

Supplementary Table 1. Calculated adsorption energies (unit in eV) for sodium polysulfide clusters on carbon supported Co_6 cluster.

cluster	adsorption energy
Na_2S_4	-4.33
Na_2S_3	-4.85
Na_2S_2	-7.85
Na_2S	-10.67
